# Promoting Well-Being in Community-Dwelling Older Adults: Effects of a Creative-Dance-Mediated Psychomotor Intervention on Life Satisfaction and Affect

**DOI:** 10.3390/nursrep16050174

**Published:** 2026-05-19

**Authors:** Hugo Rosado, Patrícia Motta, Ana Cruz-Ferreira, Catarina Pereira

**Affiliations:** 1 Departamento de Desporto e Saúde, Escola de Saúde e Desenvolvimento Humano, Universidade de Évora, Largo dos Colegiais 2, 7004-516 Évora, Portugal; hrosado@uevora.pt (H.R.); psmotta.bomfim@gmail.com (P.M.); anacf@uevora.pt (A.C.-F.); 2 Departamento de Desporto e Saúde, Escola de Saúde e Desenvolvimento Humano, Comprehensive Health Research Centre (CHRC), Universidade de Évora, Largo dos Colegiais 2, 7004-516 Évora, Portugal

**Keywords:** active aging, expressive movement, nursing, physical activity, social interaction, subjective well-being

## Abstract

**Background/Objectives**: Dance-based programs have been shown to support psychological well-being in later life, yet evidence remains limited for dance-mediated psychomotor interventions. This study examined the effects of a 12-week creative-dance-mediated psychomotor intervention on life satisfaction and positive and negative affect in community-dwelling older adults. **Methods**: This 12-week non-randomized controlled trial involved 34 participants (74.6 ± 6.6 years), allocated by convenience to an experimental group (EG) or control group (CG). The EG participated in a creative-dance-mediated psychomotor intervention (3×/week; 60 min/session; 36 sessions), while the CG maintained usual daily activities. Life satisfaction was assessed using the Satisfaction With Life Scale (SWLS), and affective experience was assessed using the Positive and Negative Affect Schedule (PANAS) at baseline and post-intervention. **Results**: No adverse events occurred; attendance was 89.8%. Within-group comparisons showed significant improvements in the EG for SWLS (20.4%), PANAS positive affect (14.3%), and PANAS negative affect (−13.9%), *p* < 0.05. In the CG, a significant improvement was observed only for PANAS negative affect (−11.5%), *p* < 0.05. Post-intervention comparisons between groups revealed significant differences favoring the EG for SWLS (*p* = 0.018) and PANAS positive affect (*p* < 0.001), with no significant between-group differences at baseline. **Conclusions**: Over 12 weeks, the intervention was associated with higher life satisfaction and positive affect in the EG compared with the CG. These findings suggest that this intervention format is safe and feasible and may support psychological well-being in community-dwelling older adults.

## 1. Introduction

Older adults increasingly live longer, yet longevity alone does not guarantee good mental health and quality of life [1]. Subjective well-being is a core component of healthy aging and reflects how people evaluate and experience their lives [2]. It is commonly described in terms of life satisfaction and two affective dimensions: positive and negative affect [3,4]. Because these dimensions represent related but distinct aspects of well-being, assessing life satisfaction together with positive and negative affect provides a broader characterization of psychological well-being [5]. Importantly, evaluative measures such as life satisfaction can differ from experienced well-being, and these dimensions may show different associations with health and functioning in later life [6]. Furthermore, evaluative and affective components of well-being may show different age-related patterns [7].

Maintaining well-being may become more challenging in later life, as aging-related changes and contextual constraints may restrict participation in activities and reduce opportunities for social and emotional engagement [5]. As people age, social networks may become smaller and age-related transitions can increase vulnerability to social isolation and loneliness [8]. In community-dwelling older adults, interventions that are enjoyable, adaptable, and feasible may be particularly relevant, as sustained participation is often a prerequisite for meaningful benefits [9]. More broadly, social connection has been described as a determinant of health and longevity, and lacking social connection as a risk factor for adverse health outcomes [10].

Movement-based interventions have received increasing attention as potential strategies to support mental health in older adults, especially when they combine physical activity with social interaction and creative engagement [11]. Dance interventions are frequently described as multimodal activities integrating motor, cognitive, sensory, and social stimulation, and evidence from systematic reviews and meta-analyses suggests beneficial effects on psychological health and quality-of-life outcomes in older adults [12]. However, the evidence base remains variable in methodological quality and intervention characteristics (e.g., dance style, frequency and duration, and outcome selection), which limits direct comparisons across studies. Dance-based interventions may also achieve high adherence and completion rates, supporting sustained participation in community settings [12]. Beyond physical benefits, dance participation has been associated with improvements in mood and social interaction, which may be particularly relevant for affective well-being in older adults [13]. In line with this rationale, creative dance interventions in older adults have been associated with improvements in life satisfaction [14], and dance-based interventions have also been linked to favorable trends in affective outcomes, including higher positive affect and lower negative affect [15].

Psychomotor interventions are typically described as a therapeutic approach addressing three dimensions, motor, emotional–affective, and cognitive, through playful, goal-directed movement activities and bodily experiences [16,17]. This emotional–affective dimension may be particularly relevant when examining changes in positive and negative affect. Within movement-based approaches, creative dance offers a particularly promising option. When delivered as part of a psychomotor-based intervention, it encourages body awareness and movement exploration, alongside rhythm and spatial awareness and shared movement creation [17,18]. Importantly, this practice brings together core elements of psychomotor intervention and dance by integrating participant-generated movement, which can foster creativity, self-expression, and communication [14,16,19].

Despite growing interest in dance-based approaches for older adults, evidence remains limited regarding creative-dance-mediated psychomotor interventions and their effects on life satisfaction and affect. In particular, the literature does not consistently include a comparison group or report effect sizes, limiting causal inference [13]. To address this gap, this study examined changes in life satisfaction and positive and negative affect following a 12-week creative-dance-mediated psychomotor intervention.

## 2. Materials and Methods

### 2.1. Study Design and Setting

A controlled, single-blinded, non-randomized pre–post study with a quasi-experimental design was conducted over 12 weeks in Portugal. Recruitment and data collection took place between March 2019 and July 2020. The participants completed assessments at baseline and immediately after the intervention period. The study procedures were reported in accordance with the SPIRIT 2013 Statement, and the protocol was registered at ClinicalTrials.gov (NCT04311931).

### 2.2. Participants

Community-dwelling older adults were recruited through leaflets/flyers, verbal invitations, and brief presentations in community settings commonly attended by older people (e.g., senior universities and recreational/community centers). Eligible participants met the following criteria: (i) age ≥ 60 years; (ii) absence of cognitive impairment, screened with the Clock Drawing Test (score > 18) [20]; (iii) independent living in the community and independent mobility; (iv) no physical limitations that would compromise participation in the sessions; and (v) no participation in dance (or similar structured dance-based interventions) within the previous 12 months.

**Figure 1:**
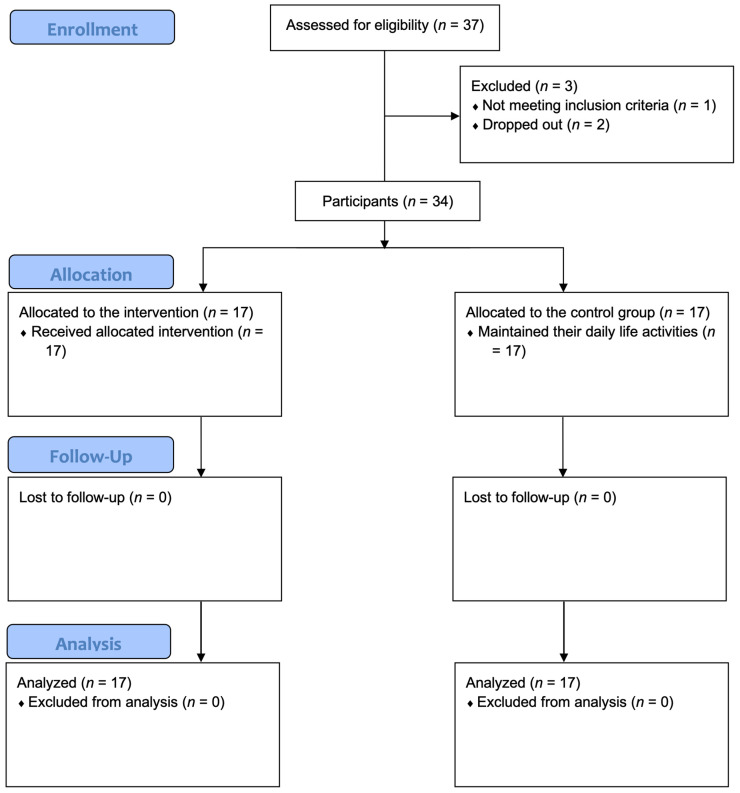
A flow diagram of the study participants.

Allocation to the experimental group (EG) or control group (CG) was performed by convenience due to practical constraints typical of community-based interventions (e.g., participants’ availability and proximity to the intervention location). Because of the non-randomized, community-based nature of the study, allocation concealment was not feasible. To enhance comparability, allocation procedures aimed to balance groups at baseline on key characteristics such as age, sex, and educational level. A total of 37 volunteers were assessed for eligibility; three did not meet the inclusion criteria and were excluded. The remaining 34 participants were allocated to the EG (*n* = 17; 15 females, 2 males) or CG (*n* = 17; 16 females, 1 male). All participants completed the study (Figure 1). Given the community-based setting and feasibility constraints, no formal a priori sample size calculation was performed; the sample size reflects the number of eligible participants recruited within the study period.

The CG maintained usual routines during the study period and reported no participation in structured exercise programs or dance classes. After study completion, CG participants were offered the opportunity to attend a similar intervention. All the participants provided written informed consent prior to inclusion. The study was conducted in accordance with the Declaration of Helsinki and was approved by the University of Évora Ethics Committee (reference 16012).

### 2.3. Procedures

Assessments were performed individually at baseline and post-intervention by the same trained evaluator. The evaluator was blinded to the study objectives and participants’ group allocation. The participants and the intervention facilitator were not blinded to group allocation; however, the participants were not informed about the specific outcomes of interest or the expected direction of change. Data collection was conducted in a quiet laboratory room under standardized conditions and scheduled in the morning to minimize contextual variability. Self-report questionnaires were self-administered; when needed, the evaluator clarified item meaning without influencing responses.

### 2.4. Outcome Measures

#### 2.4.1. Life Satisfaction

Life satisfaction was assessed using the Satisfaction With Life Scale (SWLS), a 5-item self-report measure of global life satisfaction [3,21]. The items were rated on a Likert scale ranging from 1 (“strongly disagree”) to 5 (“strongly agree”), and a total score was computed by summing responses. Total scores range from 5 to 25, with higher scores indicating greater life satisfaction.

#### 2.4.2. Positive and Negative Affect

Affective experience was assessed using the Positive and Negative Affect Schedule (PANAS), a 20-item measure comprising two 10-item subscales: positive affect (PA) and negative affect (NA) [4,22]. The participants rated how they had felt during the last weeks on a 5-point Likert scale ranging from 1 (“not at all or very slightly”) to 5 (“extremely”). PA and NA scores were computed by summing their respective items. The subscale scores range from 10 to 50, with higher scores indicating higher positive affect (PA) or higher negative affect (NA); accordingly, lower NA scores reflect fewer negative affective states.

#### 2.4.3. Complementary Measures

Sociodemographic characteristics were collected through a scripted interview. Cognitive status was assessed using the Clock Drawing Test (CDT) as an eligibility screening measure [20]; the CDT is a widely used screening tool for detecting cognitive impairment in otherwise healthy individuals [23], with scores ranging from 0 (worst) to 20 (best). Body weight and height were measured using calibrated SECA equipment, and body mass index (BMI) was calculated (kg/m^2^).

### 2.5. Psychomotor Intervention Mediated by Creative Dance

The participants allocated to the EG attended a psychomotor intervention mediated by creative dance delivered over 12 weeks (3 sessions/week, 60 min/session, 36 sessions). The sessions were group-based and conducted in the gerontopsychomotricity laboratory. The intervention was planned and led by the study’s second author, who holds an academic degree in dance and psychomotricity, and sessions were delivered using a consistent structure and progressive task progression throughout the intervention.

The intervention aimed to promote body awareness (e.g., breathing cues and awareness of body parts), as well as spatial and temporal awareness (e.g., directions, trajectories, levels, and changes in speed) and movement dynamics (e.g., variations in intensity and quality of movement). It also emphasized communication and interrelationships (paired and group-based interaction), using music as a mediator and incorporating both guided tasks and open-ended movement and expressive exploration. Progression was organized into three sequential blocks. During sessions 1–10, the emphasis was placed on attending to internal bodily sensations, with predominantly guided activities focused on breathing observation, postural awareness, and exploration of body parts, aiming to support familiarity with the setting, confidence in movement initiation, and early group cohesion. During sessions 11–20, activities increasingly targeted the temporal and rhythmic components of movement (e.g., slow/fast/pause, synchronization with the musical beat, and simple rhythmic patterns) alongside spatial exploration (e.g., formations, directions, and pathways), progressively increasing coordination and adaptation demands and supporting interpersonal interaction. During sessions 21–36, the intervention placed greater emphasis on creativity and improvisation, encouraging participants to create original movements and contribute to shared sequences, with an emphasis on autonomy, playful exploration, and social connection.

Each session followed the same structure: initial dialogue (≈5 min), global activation (≈10 min), main phase (≈20 min), choreographic phase (≈10 min), cool down (≈10 min), and final dialogue (≈5 min). In the initial dialogue, the participants briefly recalled what had been performed in the previous session, were informed about the aims of the current session, and were invited to report how they were feeling that day. To facilitate movement generation and communication, the facilitator used age-appropriate verbal imagery cues (i.e., simple prompts that were easy to translate into movement), and props such as scarves, balls, cones, and chairs were included to make verbal prompts more tangible and to diversify movement possibilities. The participants also created short movement “phrases” individually and in small groups (e.g., pairs or trios) and shared them with the group, supporting creativity, social interaction, and engagement. Within this phase, specific tasks were included to connect movement with affective experience (e.g., exploring and transforming tension into release, or creating movements emphasizing softness and fluidity), thereby supporting emotional expression and regulation through bodily experience. The choreographic phase involved recalling previously explored movements and co-creating new material to be incorporated into a final group choreography, reinforcing shared authorship and group identity. The cool-down phase included slower movements, breathing observation, stretching, and relaxation. In the final dialogue, the participants were invited to briefly reflect on their experience and to identify a salient sensation or take-home feeling from the session, supporting awareness of affective states in everyday life.

Music was used throughout the intervention and selected to match each session phase and participants’ preferences (e.g., classical/instrumental, Brazilian popular, and traditional Portuguese music). Attendance was monitored via an attendance sheet, and session participation was recorded at the end of each session.

A structured summary of the intervention components and progression is presented in Table 1.

### 2.6. Statistical Analysis

Data were analyzed using IBM SPSS Statistics (version 28.0) and JASP (version 0.96). Statistical significance was set at *p* < 0.05. The results are reported as mean ± standard deviation. Distributional assumptions were examined using the Shapiro–Wilk test, and homogeneity of variances was assessed using Levene’s test. As most variables did not meet parametric assumptions, non-parametric tests were applied. Within-group changes from baseline to post-intervention were assessed using the Wilcoxon signed-rank test, whereas between-group differences were examined using the Mann–Whitney U test.

Change over time was quantified as Δ (post − baseline) and, when reported, as percentage change (Δ% = [(post − baseline)/baseline] × 100). Effect size for non-parametric comparisons was calculated as r = Z/√N [24] and interpreted using Cohen’s thresholds (small = 0.10, medium = 0.30, large = 0.50) [25].

Given the small number of predefined outcomes and the study design, the results were interpreted alongside effect sizes, without formal multiple-comparison correction or covariate adjustment for potential allocation-related bias.

Missing data were examined using the MCAR test, indicating that missingness was compatible with a missing completely at random pattern. An intention-to-treat approach was applied, and missing values were imputed using the medium series method.

## 3. Results

At baseline, the experimental and control groups presented comparable sociodemographic characteristics, with no significant between-group differences in age (EG: 73.5 ± 5.9 years; CG: 75.7 ± 7.2 years), educational level (EG: 6.2 ± 2.9 years; CG: 5.3 ± 3.6 years), cognitive status (EG: 19.2 ± 0.4 points; CG: 19.2 ± 0.4 points), BMI (EG: 28.8 ± 3.4 kg/m^2^; CG: 27.5 ± 3.2 kg/m^2^), or sex distribution (*p* > 0.05). Thirty-four participants completed the study. Mean attendance across the 36 intervention sessions was 89.8%. No adverse events were reported.

**Figure 2:**
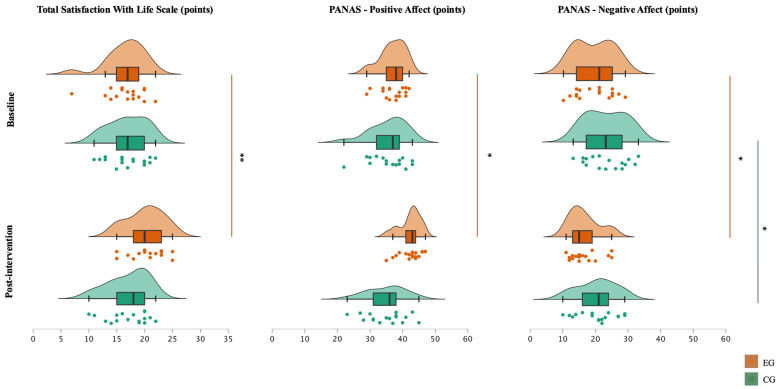
Density plots, box plots (median, interquartile range, minimum, and maximum), and jitter plots comparisons between the baseline and post-intervention evaluations; * significant differences within groups, *p* < 0.05; ** significant differences within groups, *p* < 0.001. EG: experimental group (*n* = 17); CG: control group (*n* = 17).

Descriptive data and between-group comparisons are presented in Table 2, and within-group changes are summarized in Figure 2. No significant between-group differences were observed at baseline for SWLS or PANAS outcomes. At post-intervention, the EG showed significantly higher life satisfaction (SWLS; *p* = 0.018; r = 0.41, medium) and positive affect (PANAS-PA; *p* < 0.001; r = 0.61, large) than the CG, corresponding to +17.9% higher SWLS and +21.0% higher PANAS PA in the EG at post-intervention. In contrast, post-intervention PANAS negative affect did not differ significantly between groups.

Within-group analyses indicated that the EG improved across all outcomes over the 12-week period (Figure 2): life satisfaction increased (SWLS; Δ% = + 20.4; *p* < 0.001; r = 0.62, large), positive affect increased (PANAS-PA; Δ% = +14.3; *p* = 0.001; r = 0.57, large), and negative affect decreased (PANAS-NA; Δ% = −13.9; *p* = 0.035; r = 0.36, medium). In the CG, negative affect also decreased significantly (PANAS-NA; Δ% = −11.5; *p* = 0.004; r = 0.50, large). Overall, post-intervention between-group differences were observed for SWLS and PANAS-PA, while PANAS-NA decreased in both groups.

## 4. Discussion

Overall, our findings suggest that a creative-dance-mediated psychomotor intervention may support psychological well-being in community-dwelling older adults. The EG improved in life satisfaction and in both affective dimensions, and post-intervention between-group differences favored the EG for life satisfaction and positive affect. This pattern is consistent with the notion that life satisfaction and affect represent distinct components of subjective well-being and may therefore respond differently to the same intervention [5].

Adherence is a key determinant of effectiveness in community-based interventions. In the present study, attendance across sessions was high (89.8%) and no adverse events or falls were reported, supporting feasibility and safety in this setting. This aligns with systematic evidence indicating that dance-based interventions can achieve strong adherence and completion, which is relevant for sustained participation in community contexts [9,12,13]. Comparable feasibility has also been reported in other 12-week dance trials in older adults; for example, a low-impact aerobic dance intervention reported similarly high attendance (~91%) and perceived psychological benefit [26].

The improvement in life satisfaction in the EG, together with the post-intervention between-group difference, suggests a positive effect on the life evaluation component of well-being. Improvements in life satisfaction have been reported following creative dance interventions in older adults [14]. Early community-based studies suggested potential well-being benefits of creative dance and movement programs [27], but directly comparable controlled studies with standardized outcomes remain limited [13]. Although effect-size metrics differ across studies, the improvement in life satisfaction observed here reached medium-to-large magnitudes, whereas prior creative dance trials have reported small-to-moderate effects for life satisfaction outcomes [14]. This difference may relate to variations in intervention content and delivery, including the therapeutic psychomotor approach used in the present intervention.

Positive affect increased in the EG and differed between groups at post-intervention, with a large effect size. This result aligns with systematic evidence indicating that therapeutic and other dance interventions can improve psychological outcomes in older adults [11,12,13]. Studies using PANAS measures have also reported favorable changes in affect in controlled designs. In a large quasi-experimental study with a comparison group, a 12-week dance/movement therapy intervention was associated with substantial improvements in PANAS positive mood relative to controls [28]. A meta-analysis update of controlled dance and dance/movement therapy studies reported overall medium effects on health-related psychological outcomes [29]. Although effect-size metrics differ across studies, the large post-intervention effect observed here for positive affect is broadly consistent with evidence that affect-related benefits can be moderate on average, while varying across interventions and designs [29]. In addition, dance places combined sensorimotor and cognitive demands, and training has been linked to changes in action perception, which may support broader psychological benefits [30]. Qualitative studies of creative dance also offer explanations for these changes, including enjoyment, social connection, creativity, and opportunities for autonomy [31].

From a practical perspective, improvements in life satisfaction and positive affect in the EG were not only statistically significant but also of moderate-to-large magnitude. Life satisfaction increased by approximately 20% and positive affect increased by approximately 14% over 12 weeks, suggesting potentially meaningful short-term improvements in perceived well-being. In real-world terms, higher SWLS and PA scores may reflect a more positive appraisal of daily life and more frequent positive emotional states. Larger trials are needed to confirm these effects and clarify thresholds for meaningful change in community-dwelling older adults.

Negative affect decreased significantly in both groups. In the EG, this reduction is compatible with the emotional–affective focus commonly described in psychomotor approaches and with creative dance formats emphasizing body awareness and embodied experience [16,17,18]. Negative affect also decreased in the CG, suggesting that time-related or participation-related influences may have contributed (e.g., increased self-reflection during assessments and repeated measurement). In addition, the CG started with higher negative affect, which may have allowed greater reductions over time, consistent with regression-to-the-mean effects. Because PANAS ratings referred to the “last weeks,” negative affect may have been sensitive to short-term fluctuations in daily stressors and routines. Seasonal timing may have played a minor role; however, evidence for robust seasonal shifts in mood and affect is mixed, with some studies reporting only small seasonal differences in PANAS scores and others emphasizing heterogeneity and inconsistent patterns across designs and outcomes [32,33]. Positive and negative affect can change independently, which may help explain why between-group differences were clearer for positive affect than for negative affect [34].

Overall, direct comparisons with previous studies remain constrained because relatively few studies have examined this specific intervention format with standardized psychological outcomes, and designs vary widely [13]. In addition, related psychomotor dance approaches have been described in older clinical populations (e.g., care-home settings), but differences in context and participant profiles limit direct comparison with community-dwelling samples [35].

### Limitations

Some limitations should be considered when interpreting these findings, particularly regarding generalizability. The non-randomized design and convenience allocation may have introduced selection and allocation bias despite baseline comparability (e.g., participants with higher motivation may have been more likely to enter the intervention group), and unmeasured baseline differences cannot be excluded; therefore, causal inference is limited and findings should be interpreted as preliminary and non-causal. The modest sample size and predominance of female participants limit statistical power (particularly for small effects), which may increase the risk of Type II error, reduce the robustness of estimates, and constrain generalizability, particularly to older men. In addition, the CG did not receive an attention-matched intervention, so non-specific factors such as social interaction and expectancy effects may have contributed to the observed effects; future studies should include an active control condition. Outcomes relied on self-report measures, and assessments were limited to baseline and post-intervention, highlighting the need for follow-up measurements to examine durability.

## 5. Conclusions

Over 12 weeks, the creative-dance-mediated psychomotor intervention was associated with improvements in life satisfaction and positive affect in the intervention group, while negative affect decreased over time in both groups. These results provide preliminary support for this type of intervention, which integrates creativity, self-expression, and communication in socially engaging dance-based sessions, as a feasible approach to support psychological well-being in later life. Larger studies, ideally using randomized controlled designs and including follow-up assessments, are needed to confirm these findings.

## Figures and Tables

**Table 1:** Summary of the creative-dance-mediated psychomotor intervention.

Domain	Description
Setting	Group-based sessions in a gerontopsychomotricity laboratory.
Duration and frequency	12 weeks; 3 sessions/week; 60 min/session; 36 sessions.
Goals (core targets)	Body awareness; spatial/temporal awareness; movement dynamics; communication and interpersonal interaction.
Progression	Sessions 1–10: guided body awareness; 11–20: rhythmic/spatial emphasis; 21–36: creativity/improvisation and co-creation of shared sequences/final choreography.
Session structure (≈60 min)	Initial dialogue (≈5 min); global activation (≈10 min); main phase (≈20 min); choreographic phase (≈10 min); cool down (≈10 min); final dialogue (≈5 min).
Main-phase strategies	Participant-generated movement, verbal imagery cues, and affect-linked tasks.
Materials/props	Scarves, balls, and chairs used to facilitate communication and diversify movement exploration.
Music	Used throughout and selected to match each session phase and participants’ preferences.

**Table 2:** Descriptive results of the participants’ SWLSs and PANAS scales.

Variables	Baseline (Mean ± SD)	Δ (Post-Baseline) (Mean ± SD)
SWLS (points)		
EG	16.7 ± 3.5	3.4 ± 2.6 ^a^
CG	17.0 ± 3.4	0.1 ± 1.2
PANAS—Positive Affect (points)		
EG	37.0 ± 3.8	5.3 ± 4.4 ^b^
CG	35.6 ± 5.6	−0.6 ± 3.8
PANAS—Negative Affect (points)		
EG	19.4 ± 5.8	−2.7 ± 4.7
CG	23.1 ± 6.2	−2.6 ± 3.1

SWLS: Satisfaction With Life Scale; PANAS: Positive and Negative Affect Schedule scale; SD: standard deviation; EG: experimental group (*n* = 17); CG: control group (*n* = 17); ^a^ significant differences between EG and CG, *p* < 0.05; ^b^ significant differences between EG and CG, *p* < 0.001.

## Data Availability

The data used and/or analyzed during the current study are available on request from the corresponding author due to ethical and privacy restrictions.

## Public Involvement Statement

No public involvement in any aspect of this research.

## Guidelines and Standards Statement

This manuscript was drafted against the TREND Statement (Transparent Reporting of Evaluations with Nonrandomized Designs) for non-randomized evaluation research. The study protocol was reported in accordance with the SPIRIT 2013 Statement (Standard Protocol Items: Recommendations for Interventional Trials).

## Abbreviations

The following abbreviations are used in this manuscript:

EG	Experimental group
CG	Control group
SWLS	Satisfaction With Life Scale
PANAS	Positive and Negative Affect Schedule
PA	Positive affect (PANAS subscale)
NA	Negative affect (PANAS subscale)
CDT	Clock Drawing Test
BMI	Body mass index
SD	Standard deviation
